# Reasons for Relapse After In-Patient De-Addiction Treatment for Alcohol Dependence – a Qualitative Analysis From India

**DOI:** 10.1192/bjo.2022.246

**Published:** 2022-06-20

**Authors:** Pavithra Ethirajan, Manjula Simiyon, Pradeep Thilakan

**Affiliations:** 1 Pondicherry Institute of Medical Sciences, Pondicherry, India; 2 Betsi Cadwaladr University Health Board, Wrexham, United Kingdom

## Abstract

**Aims:**

To explore the reasons for relapse after receiving in-patient detoxification and de-addiction treatment for alcohol dependence syndrome, through in-depth interviews and thematic analysis.

**Methods:**

This study was conducted in a tertiary care teaching hospital in South India. After obtaining Institutional ethics committee approval, patients of 18 years and above, who were admitted for the management of alcohol withdrawal syndrome, were approached and informed consent was obtained. Patients were screened with Clinical Institutes Withdrawal Assessment-Alcohol Revised (CIWA-Ar) and 15 patients who scored less than 7, who did not have any severe medical or psychiatric illness, whose cognition was intact according to Hindi Mental Status Examination (HMSE) and those who had two de-addiction treatments in the past were recruited. In-depth interviews were conducted in Tamil, audio-recorded, and transcribed. A semi-structured guided interview format was used to gather their narratives. The transcripts were translated to English on the same day and a step-by-step thematic analysis recommended by Braun et al was followed. The interviews were conducted in a soundproof room ensuring privacy and confidentiality. The recorded audios and the transcripts were firewall protected. The transcripts were read multiple times to familiarize the investigators. By using a general inductive method the data were retrieved, coded, and systematically organized according to patterns and themes. Two investigators coded the transcripts separately and any conflict was resolved by discussion. Thematic saturation was attained with the 14th transcript but the coding was completed for all 15 manuscripts. The mean age of the participants was 26.4 years.

**Results:**

The analysis resulted in the identification of reasons attributed by the patients for resuming drinking after receiving in-patient detoxification and de-addiction treatment for 21 days. This 21 days deaddiction program comprises of detoxification, motivation enhancement therapy, group therapy, and family interventions. The reasons for relapse included peer pressure, confidence that they will not become dependent again, craving, stressors, and health issues such as pain and insomnia and to test whether the treatment works or not. Reasons for the delay in help-seeking were lack of motivation, poor social support, financial constraints, lack of hope in medical treatment, did not feel the necessity to take treatment, fear of whether the doctors would be upset for relapsing again and, the guilt of letting down the treatment team. The reasons why they finally came for treatment were having severe withdrawal symptoms, pressure from a family member or employee, guilt and a desire to change, and fear of dying.

**Conclusion:**

This research provides avenues to understand patients’ perspectives on relapse of alcohol dependence. Understanding these would be beneficial in psychotherapy while managing relapses. It also helps us to reflect on our practice and to address these issues before discharging the patients to minimize the relapses.

